# Reprogramming tumor immunity through APOBEC3s-mediated mutagenesis: from genome instability to immune checkpoint interactions

**DOI:** 10.3389/fimmu.2026.1765368

**Published:** 2026-02-11

**Authors:** Qiaoxi Li, Wenyu Wan, Zihan Zhu, Xuanduo Lin, Fei Wang, Mengmeng Li, Hao Guo, Yang Yang

**Affiliations:** 1 Department of Dermatology, The First Hospital of China Medical University, Shenyang, China; 2 Key Laboratory of Immunodermatology, Ministry of Education, and National Health Commission, National Joint Engineering Research Center for Theranostics of Immunological Skin Diseases, Shenyang, China

**Keywords:** APOBEC3 cytidine deaminases, genomic instability and DNA repair, immune checkpoint blockade, mutational signatures, neoantigens and immunotherapy resistance, precision immuno-oncology, tumor immune microenvironment, tumor immunoediting

## Abstract

The apolipoprotein B mRNA editing catalytic polypeptide-like (APOBEC) family was first defined as an innate antiviral defense system, but the APOBEC3 subfamily (APOBEC3s) is now recognized as a major endogenous source of somatic mutagenesis in cancer. APOBEC3s enzymes, particularly APOBEC3A and APOBEC3B, generate characteristic mutation patterns that promote genomic instability, clonal evolution, and adaptation to therapy. Beyond driving tumor evolution, APOBEC3 activity reshapes antitumor immunity in solid cancers. APOBEC3-induced mutations increase tumor mutational burden and create neoantigens that can enhance CD8^+^ T-cell infiltration and interferon signaling. However, sustained APOBEC activation may also reinforce immunosuppressive circuits: through chronic inflammation and PD-1/PD-L1–interferon signaling, tumors can induce T-cell dysfunction, immune escape, and resistance to immune checkpoint blockade. This functional ambivalence has sparked debate over whether APOBEC3s should be inhibited to limit genomic instability, leveraged to enhance tumor immunogenicity, or modulated dynamically in a context-dependent manner. This review outlines the immune landscape and biochemical characteristics of the APOBEC3 family and situates these features within broader cancer-related disease contexts. APOBEC3-mediated mutagenesis is discussed as a mechanistic link between genomic instability and tumor–immune crosstalk in solid tumors, with emphasis on its relationships to immunoediting, immune checkpoint pathways, and therapeutic responses. Context-dependent associations of APOBEC3 activity with immune activation or immune evasion are also considered, together with their implications for strategies that modulate this pathway.

## Graphical abstract

**Figure:**
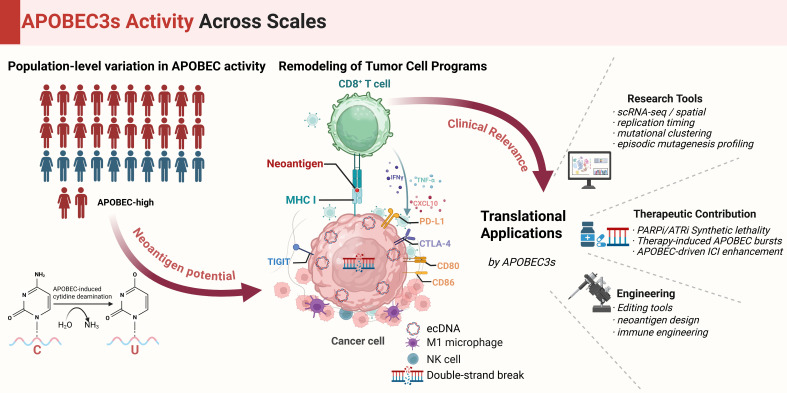

## 1 Introduction

The apolipoprotein B mRNA editing catalytic polypeptide-like (APOBEC) family is a group of cytidine deaminases that act on single-stranded polynucleotides. These enzymes convert cytosine (C) to uracil (U) and thereby change the sequence of DNA or RNA (1). Early studies on apolipoprotein B mRNA editing have shown that this reaction does not require additional nucleotides or cofactors. Subsequent work expanded this view by showing that several APOBEC family members also have DNA deaminase activity. These enzymes can inhibit the replication of retroviruses such as HIV-1 during infection (1, 2). These findings established APOBEC proteins not only as RNA-editing enzymes but also as key antiviral factors within the innate immune system. Among the family members, the APOBEC3 subfamily (APOBEC3s) has attracted particular attention because of its robust activity on both viral and cellular DNA.

With continued investigation, APOBEC3s is viewed as endogenous sources of mutagenesis that connect host antiviral defense with tumor-associated genetic alteration (3, 4). During viral infection, APOBEC3s induces mutations in viral genomes and thereby interferes with viral replication. However, when its deaminase activity accidentally targets the host genome, APOBEC3s can cause DNA point mutations and contribute to genomic instability and tumor-related hypermutation (3, 5).

It is also important to note that deamination-based editing is not inherently detrimental. Controlled DNA modification and repair are essential for normal immune responses, antibody affinity maturation, and cellular development (4, 6). In cancer, however, APOBEC3 enzymes act as major drivers of tumor mutational burden. Off-target mutations may activate oncogenes or inactivate tumor suppressor genes, thereby promoting tumorigenesis. At the same time, these mutations may increase neoantigen load, enhancing immune recognition and elimination of tumor cells. This double-edged role makes APOBEC3s a crucial molecular node that connects virology, immunology, and oncology (4, 7).

In summary, the APOBEC family functions not only in antiviral defense but also in shaping tumorigenesis, mutational landscapes, and tumor immunogenicity. This review focuses on APOBEC enzymes with particular emphasis on APOBEC3s and covers their family composition, enzymatic properties, and roles in genome mutagenesis. Recent advances defining APOBEC3 functions in tumor immunology are also discussed.

## 2 The APOBEC family landscape and functional diversity

The human genome encodes 11 APOBEC family genes, grouped into five subfamilies: activation-induced deaminase (AID) and APOBEC1–4 (8). AID and APOBEC1 are both located on chromosome 12, whereas APOBEC2 is located on chromosome 6. The APOBEC3s gene cluster consists of seven tandemly arranged members, A3A–A3D and A3F–A3H, on chromosome 22. APOBEC4 is located on chromosome 1 (9, 10). This genomic organization provides the structural basis for the functional diversity of the family.

These genes show clear tissue specificity. AID is mainly expressed in activated B cells and mediates somatic hypermutation and class-switch recombination of immunoglobulin genes (11, 12). APOBEC1 is highly expressed in the liver and small intestine, where APOBEC1 edits ApoB-100 mRNA and regulates lipid metabolism and cholesterol homeostasis (13, 14). APOBEC2 is expressed primarily in the cardiac and skeletal muscles. Although its catalytic activity has not been fully established, available evidence links APOBEC2 to muscle development and DNA-binding functions (15, 16). Regulated by PAX7 and acting through DNMT3B-dependent DNA methylation, APOBEC2 helps maintain myogenic transcriptional programs and muscle homeostasis, whereas its loss or dysregulation leads to global DNA hypermethylation, defective myogenesis, and myopathic changes. APOBEC4 is expressed predominantly in the testis and has been proposed to participate in ribosome biogenesis (17). The molecular functions and substrate specificities of APOBEC2 and APOBEC4 remain incompletely defined (18).

APOBEC3s represents a more evolutionarily advanced branch of the APOBEC system. APOBEC3 genes emerged in placental mammals, broadening the antiviral repertoire of the family (19). APOBEC3 proteins are widely expressed in peripheral blood leukocytes, and APOBEC3A (A3A) and APOBEC3G (A3G) are the most abundant members (20, 21). Functionally, these enzymes contribute to innate immunity by restricting retroviral replication, including HIV-1, and by suppressing endogenous retroelements such as Long Interspersed Nuclear Element-1 and Alu short interspersed nuclear elements (Alu SINEs) (22, 23). Through these activities, APOBEC3s provides a mechanistic link between intrinsic cellular defense and the maintenance of genome stability.

Subcellular localization further shapes the function of each APOBEC3 member. A3A, APOBEC3B (A3B), APOBEC3C (A3C), and APOBEC3H (A3H) are mainly localized in the cytoplasm (7, 9). A3G is enriched in the cytoplasm but can also localize partly in the nucleus. APOBEC3F (A3F) is primarily cytoplasmic, and its distribution may be modulated by other family members. These enzymes usually exist as monomers or oligomers. Oligomerization changes their binding affinity for single-stranded DNA or RNA and modulates their catalytic activity, and this regulation shapes their overall mutagenic potential (24–26).

During HIV-1 infection, A3G and A3F can be packaged into viral particles. After entry into a new host cell, these enzymes deaminate cytidines to uridines in nascent viral cDNA, generating C-to-U (G-to-A on the opposite strand) mutations that impair viral replication. To counter this restriction, the HIV-1 accessory protein Vif binds APOBEC3 proteins and recruits ubiquitin–proteasome machinery for their degradation, thereby preventing their packaging and activity (22, 24). This antagonistic interplay has become a canonical example of APOBEC–virus coevolution.

All AID/APOBEC family members share a common enzymatic core. Each protein contains one or two zinc-dependent deaminase domains with a conserved histidine–X–glutamic acid–X23–28–proline–cysteine–X2–4–cysteine motif, where X represents any amino acid (27, 28). In the catalytic center, histidine and cysteine residues coordinate a Zn^2+^ ion, and the glutamate residue participates in proton shuttling (29, 30). Together, these residues generate an activated hydroxide ion that catalyzes the deamination reaction. This conserved catalytic mechanism provides a common biochemical foundation for the diverse biological effects observed across APOBEC family members.

APOBEC proteins recognize specific sequence motifs and deaminate cytosines, leading to C-to-T or C-to-G mutations. Each family member has its own preferred substrates and biological roles (31–33). A3A preferentially targets the TTC motif (31). A3B prefers DNA hairpin structures, but the mutation spectrum it induces differs from that of A3A (32). A3G specifically recognizes 5′-CC sites and especially favors the deamination of the third cytosine in such contexts; the flexible structure of loop 7 in A3G is a key determinant of this recognition (6, 24). These differences give rise to distinct APOBEC-associated mutational signatures in human cancers. Representative mutation motifs and patterns are summarized in **Table 1**. APOBEC activity, therefore, not only contributes to viral clearance but also shapes the mutational landscape of human cancers.

**Table 1:** Representative APOBEC-associated mutational motifs and patterns.

APOBEC3 member	Substrate preference	Preferred motif	ssDNA source	Nuclear/cytosolic localization	Mutagenic strength	Unique features	References
A3A	RNA/ssDNA	TTC; TpCpN	Replication/transcription ssDNA	Predominantly nuclear	High	DSB induction; ecDNA; kataegis	(3, 32, 34–36)
A3B	ssDNA	TCA/TCT; hairpin DNA	Lagging-strand ssDNA	Nuclear	Medium–high	Hairpin DNA	(21, 32, 35, 37, 38)
A3C	ssDNA	TCA/TCT/CCA/CCT/CCC	Transcription-exposed ssDNA	Nuclear/cytosolic	Low	Mild mutator	(20, 22, 39)
A3G	RNA/ssDNA	5′-CC (3rd C preferred)	Reverse-transcription ssDNA	Cytosolic	Medium	DSB repair	(6, 22, 24, 40, 41)

DSB, double-strand break; ecDNA, extrachromosomal DNA.

Deamination can also lead to more severe genomic consequences. Under certain contexts, AID/APOBEC-mediated deamination produces nonsense mutations such as TGG-to-TAG changes (42, 43). Such mutations can inhibit viral replication or cause loss-of-function alterations in host genes. Expression patterns further reinforce the link between APOBEC3s and cancer. APOBEC1, APOBEC2, APOBEC4, and AID show relatively restricted tissue expression.

In contrast, APOBEC3 proteins are expressed at low levels in many normal tissues but are often strongly upregulated in tumors (44, 45). This pattern links APOBEC3s closely to the initiation and progression of human cancers. Researchers now recognize a dual role for APOBEC proteins. APOBEC activity can promote tumor evolution through mutation while also enhancing antitumor immunity by generating neoantigens. This functional duality—driving tumor evolution while enhancing immunogenicity—provides a conceptual framework for leveraging APOBEC-associated mutagenesis to augment tumor recognition in cancer immunotherapy.

## 3 APOBEC3-mediated mutagenesis in tumor evolution

Tumor initiation and progression are usually driven by oncogene activation or tumor suppressor gene inactivation, and defects in DNA damage and repair pathways act as key determinants of many mutational patterns (4, 46, 47). Within this context, the APOBEC3 deaminase family has emerged as an important endogenous mutational driver. Approximately 60% of tumor samples show APOBEC-associated mutational features, which are particularly prominent in bladder cancer, head and neck squamous cell carcinoma (HNSCC), breast cancer, and lung cancer (48). The C→T and C→G mutations that APOBEC3s generates have been defined as distinct mutational signatures, such as SBS2 and SBS13 (49–51). Notably, APOBEC activity has been linked to recurrent driver hotspot mutations in specific cancer genes. For example, the activating PIK3CA helical-domain hotspots E542K and E545K map to APOBEC-preferred TCW motifs (on the opposite strand) and are enriched in APOBEC-high or HPV-associated tumors, supporting APOBEC-mediated cytidine deamination as a plausible source of these oncogenic events (52). Similarly, in bladder cancer, the common activating FGFR3 S249C hotspot represents an APOBEC-type motif; tumors harboring S249C show enrichment of APOBEC mutational signatures and increased AID/APOBEC family expression, and *in vitro* deamination assays confirmed S249 as an APOBEC target (53). These APOBEC3-driven changes promote genomic instability and clonal diversity and thereby support tumorigenesis and adaptive evolution.

**Figure 1:**
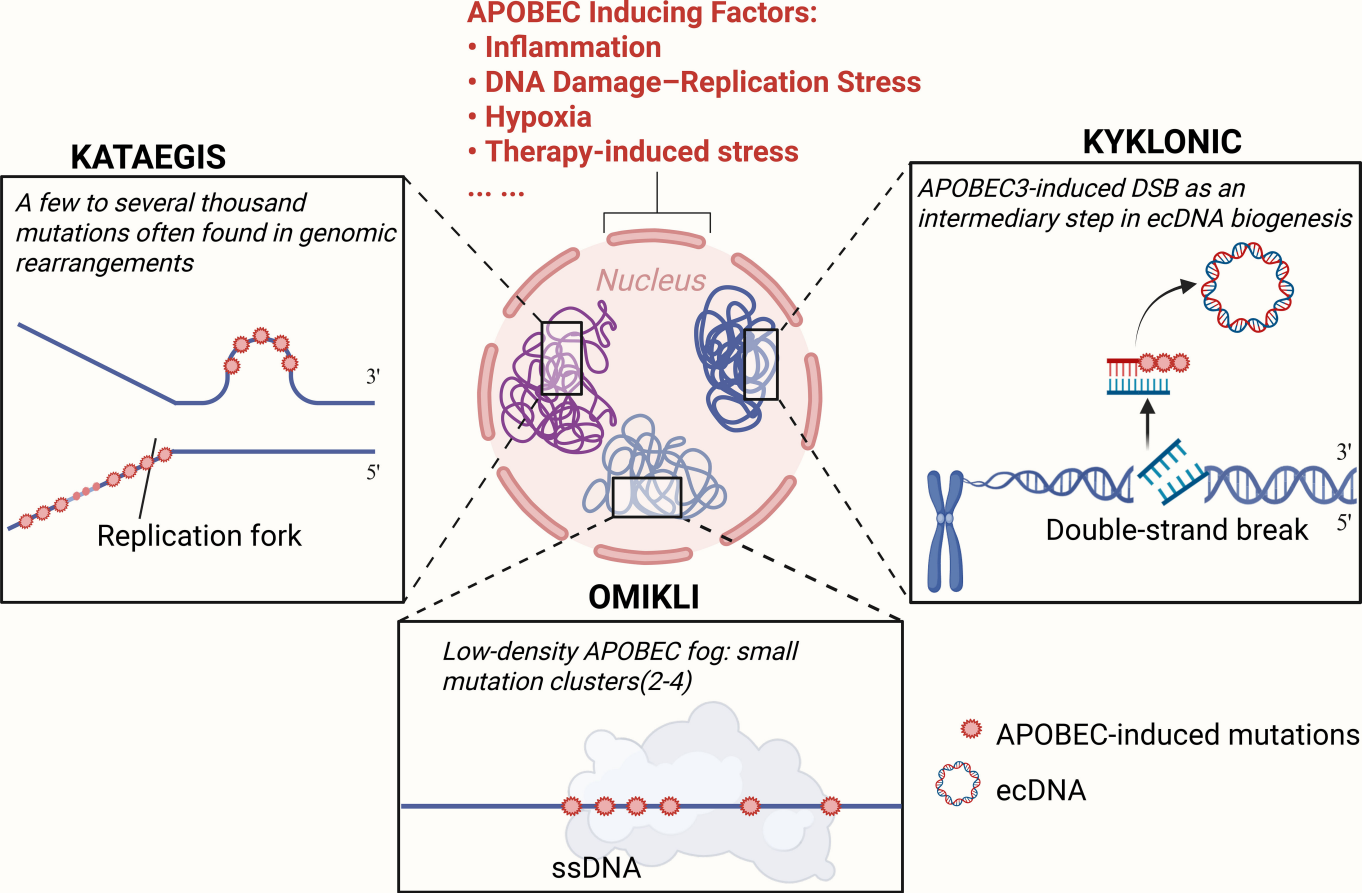
Distinct patterns of APOBEC3-driven mutagenesis in cancer genomes. External cues—including inflammation, DNA damage/replication stress, hypoxia, and therapy—can activate nuclear APOBEC3 enzymes. Depending on context and ssDNA availability, APOBEC mutagenesis is often described in three patterns: kataegis, dense localized mutation showers often tracking replication and enriched near rearrangements; omikli, sparse small clusters (typically two to four mutations) scattered across exposed ssDNA; and kyklonic, broader damage linked to APOBEC-associated double-strand breaks that may contribute to ecDNA formation and clustered APOBEC-type mutations on circular amplicons. The figure was created with BioRender. ecDNA, extrachromosomal DNA.

APOBEC3s-induced deamination tends to occur at cytosines within TpCpN motifs or their complementary NpGpA sequences and creates characteristic mutational marks (12, 51). This mutational pattern is highly stereotyped. Whole-exome sequencing studies have shown that dysregulated APOBEC activity can generate widespread single-nucleotide variants and insertions or deletions in cancer genomes (50, 54, 55). These changes often cluster into localized hypermutation events known as kataegis. Multiple APOBEC3-driven mutational patterns have been described so far, including kataegis, omikli, and kyklonic configurations, and are summarized in **Figure 1**. When APOBEC3s encounters single-stranded DNA exposed at replication forks or transcription bubbles or during DNA double-strand break repair, they can slide along the exposed strand and induce consecutive mutations (56).

Work in human gene therapy has provided direct evidence of this process. In lentiviral vectors used for patients with adenosine deaminase–severe combined immunodeficiency, duplex sequencing identified abundant G→A mutations that match APOBEC3s signatures (57). This finding further suggests that APOBEC3s activity is not limited to viral RNA or infection-associated single-stranded DNA. APOBEC3s can also catalyze C→U deamination on cellular single-stranded DNA transiently exposed during DNA replication or reverse transcription. Collectively, these observations support a mutagenic role for APOBEC3s in replication- and retrotranscription-linked processes and underscore the persistent mutational potential of these enzymes as host defense factors.

Several APOBEC3 members, including A3A, A3B, and A3G, are now annotated as cancer-associated genes (58). A3B expression shows a strong association with higher APOBEC3s-induced mutational burden, while A3A, A3F, and A3G display weaker but still positive correlations (45). Notably, the frequencies of A3A- and A3B-specific mutational signatures do not scale linearly with their mRNA expression levels (37, 56, 59, 60). This observation suggests that multiple layers of regulation, including protein stability, post-translational modifications, substrate utilization efficiency, and repair of deaminated dU, all shape their mutagenic output. Experimental studies have further shown that overexpression of A3A or A3B induces marked genomic instability in breast and other epithelial cancer cells (55, 61). Transient high-level A3A expression is well established to drive mutation accumulation in the nuclear genome, and available evidence further suggests that mitochondrial DNA can be targeted in parallel under such conditions (62). Nested PCR/3DPCR and sequencing have shown that only a small fraction of mitochondrial genomes in patient tissues and UNG-deficient cells is massively hypermutated at APOBEC3-signature TpC/CpC sites, whereas most mtDNA remains unedited. These hyperedited mtDNA molecules are thought to accumulate in the cytoplasm and be channeled into UNG-dependent DNA catabolism rather than maintained as functional genomes, thereby providing danger signals in parallel with APOBEC3-driven nuclear DNA damage (63). This process provides a chronic source of genotoxic and inflammatory stress that, particularly in settings of long-standing inflammation such as chronic hepatitis, may foster clonal selection and cancer evolution.

Transient bursts of APOBEC3s-induced hypermutation appear to be key events in tumor evolution. These bursts can generate multiple mutations within a single cell over a short period without requiring a long-term high-mutator state (40, 64, 65). This process increases intratumoral heterogeneity and subclonal diversity and provides the genetic basis for clonal selection and the emergence of drug-resistant clones. After APOBEC3s-mediated cytosine deamination, the resulting uracil should be removed by uracil-DNA glycosylase and the base excision repair pathway (37). If repair fails, these lesions become fixed as permanent mutations. When DNA mismatch repair, non-homologous end joining (NHEJ), or homologous recombination (HR) is impaired, APOBEC3-induced DNA damage cannot be efficiently resolved. This failure leads to severe genomic instability and can cause cell death. Such dependency forms a classical synthetic lethality relationship and ultimately contributes to the emergence of a mutator phenotype in tumors (34, 66, 67).

Further studies have indicated that APOBEC3s-mediated deamination not only generates point mutations but can also directly cause DNA double-strand breaks and promote structural genome rearrangements. Upregulation of A3A can directly trigger double-strand breaks. In addition, APOBEC3s-induced “kyklonic” clustered mutations are detectable in approximately 69% of structural variants that give rise to extrachromosomal DNA (ecDNA), and these mutations are highly enriched near breakpoint regions (34). These findings suggest that APOBEC3s-induced double-strand breaks may represent an important intermediate step in ecDNA formation. This process can create circular amplicons that carry oncogenes such as CCND1 and thereby accelerate tumor heterogeneity and chemoresistance.

Recent whole-genome sequencing studies have also uncovered spatial coupling between APOBEC3s-induced mutations and changes in DNA replication timing. In breast cancer and lung adenocarcinoma, approximately 6%–18% of the genome shows altered replication timing (68). Genomic regions that shift from late to early replication are preferentially enriched for APOBEC3s-mediated localized hypermutation, known as omikli events, and frequently contain driver mutations in genes such as *ERBB2* and *ESR1* (68). These replication timing shifts are closely associated with defective mismatch repair. Together, these observations link APOBEC3-driven genomic instability to replication-associated remodeling and to early stages of tumor evolution.

In tumors with homologous recombination defects, APOBEC3s-induced mutation rates are even higher (37). High mutational burden often correlates with greater aggressiveness and resistance to standard therapies (69). Several converging mechanisms appear to drive this enhanced APOBEC3s mutagenesis (35, 56, 67, 70–72). Replication stress and defective DNA repair promote the accumulation of single-stranded DNA, which provides abundant substrates for APOBEC3s enzymes. Cancer cells that experience chronic genomic instability or specific oncogenic events, therefore, face persistent replication stress and can continue to acquire APOBEC3s-driven mutations during later stages of tumor evolution. At the same time, the ability of APOBEC3s proteins such as A3A, A3B, and APOBEC3D to enter the nucleus through nuclear localization signals or multiprotein complexes allows them to act directly on nuclear DNA and to influence cell-cycle profiles (21). Inflammatory cues and cellular stress further amplify this effect. Cytokines and genotoxic therapies can upregulate APOBEC3s expression and induce DNA damage, which together increase both enzyme levels and single-stranded DNA substrate availability and thus reinforce the mutational output (36, 73). Cytokine-driven type I interferon signaling and genotoxic therapies (such as replication-stressing nucleoside analogues, irradiation, or platinum drugs) can transiently upregulate A3A and A3B while at the same time inducing replication stress, double-strand breaks, and base excision repair intermediates that expose long tracts of single-stranded DNA (36, 71, 74). Together, increased APOBEC3s abundance and therapy-generated ssDNA substrates amplify cytosine deamination and reinforce APOBEC mutational output, and in tumors with pre-existing high A3B expression, such as clear cell ovarian carcinoma, this cooperation with platinum-induced crosslinks can produce synergistic levels of DNA damage and shape treatment responses (36, 73).

Beyond substrate supply, transcriptional regulation is a central determinant of APOBEC3s mutagenic potential. *Her2* activation enhances cellular stress signaling and increases A3A expression. RelA and Bach1 can bind directly to the A3A promoter and modulate its transcriptional activity (75). The correlation between A3A and the interferon signaling factors STAT1 and STAT2 arises mainly from tumor-infiltrating immune cells rather than from the cancer cells themselves. This pattern suggests that A3A expression is strongly shaped by the tumor microenvironment.

Building on these insights into APOBEC3-driven genomic instability and its regulatory networks, it is crucial to understand how APOBEC3s activity intersects with the tumor immune microenvironment (TIME). We briefly summarize the key inflammatory, genomic, microenvironmental, and therapy-induced contexts that regulate APOBEC3 activity in solid tumors, along with their genomic and immunologic consequences (**Table 2**).

**Table 2:** Contextual regulation of APOBEC3 activity in cancer.

Regulatory axis	Key regulators	Mechanistic summary	Cancer types	Impact on mutagenesis/immunity	References
Inflammatory–immune signaling	IFN-γ, TNF-α; STAT1/STAT2	Cytokine-driven A3 induction	Multiple cancers	APOBEC ↑, neoantigens ↑, inflamed TIME	(16, 22, 27, 36, 75–79)
DNA damage–replication stress	Replication stress; DNA mismatch repair (MMR)/HR/NHEJ loss	ssDNA exposure ↑	Multiple cancers	Hypermutation ↑, structural variants (SVs) ↑, PARPi/ATRi sensitivity ↑	(3, 19, 35, 47, 56, 68, 69, 71)
TIME and innate immunity	cGAS–STING; hypoxia	STING activation; A3A/A3B/A3G ↑	Multiple cancers; gastric organoids	CD8^+^ T cells ↑, inflammation ↑, instability ↑	(30, 63, 74, 80–82)
Therapy-induced stress	Chemotherapy, radiotherapy, ICB	Transient A3 upregulation	Urothelial carcinoma; breast cancer; HNSCC; NSCLC	Subclonal evolution ↑; resistance ↑; ICI response ↑	(41, 61, 67, 76, 83–88)

TIME, tumor immune microenvironment; HR, homologous recombination; NHEJ, non-homologous end joining; ICB, immune checkpoint blockade; HNSCC, head and neck squamous cell carcinoma; NSCLC, non-small cell lung cancer; ICI, immune checkpoint inhibitor.

## 4 Linking APOBEC3s activity to the TIME

Polynucleotide cytidine deaminases support several diverse biological functions, from lipid metabolism to adaptive and innate immunity, and form the basis of a unique mode of adaptive immunity. This pattern suggests that APOBEC3s may be induced in the TIME as part of the host immune response (70).

Notably, deaminase-driven mutagenesis is not unique to solid tumors. In contrast to APOBEC3s-driven mutagenesis in solid tumors, AID provides a paradigmatic example of an APOBEC family deaminase driving genome instability in the hematopoietic compartment. AID dysregulation contributes to B-cell malignancies through off-target deamination and translocation-prone DNA breaks (89, 90). Recent whole-genome analyses further showed that super-enhancers can be hypermutated in diffuse large B-cell lymphoma with patterns consistent with AID activity, linking regulatory element mutagenesis to altered oncogene programs (91). Collectively, these observations highlight a shared principle: AID operates within programmed antibody diversification in B cells, whereas APOBEC3s is broadly inducible and frequently coupled to replication stress and inflammatory cues in solid tumors.

At the molecular level, APOBEC3s-induced mutations can generate neoantigens (neoepitopes), such as CSDE1-derived heteroclitic neoepitopes, and these mutant peptides often show altered peptide properties, including increased hydrophobicity, which may influence antigen presentation and immune recognition (92, 93). Accordingly, APOBEC3s-associated mutational signatures correlate with higher immunogenicity, more active CD8^+^ T-cell infiltration, and IFN-γ signaling activation and can extend patient survival (94, 95).

At the cellular level, in HNSCC and breast cancer, APOBEC3s-associated mutational burden positively correlates with the tumor cell cytolytic score, which suggests that tumors with high APOBEC3s-associated mutational burden have active T-cell signaling pathways (76, 94). A3A activity can enhance the accumulation of cytosolic double-stranded DNA and activate the cGAS–STING pathway, thereby inducing antitumor CD8^+^ T-cell responses. In esophageal squamous cell carcinoma (ESCC), enforced A3A expression induces extensive nuclear DNA damage and double-strand breaks, which promote leakage of nuclear-derived double-stranded DNA into the cytosol. This cytosolic dsDNA accumulates and colocalizes with cGAS, leading to STING–TBK1–IRF3 activation, upregulation of type I interferon-stimulated genes and chemokines such as CXCL10 and CCL5, and ultimately enhanced recruitment and activation of granzyme B^+^ CD8^+^ T cells, consistent with an immune-inflamed transcriptional and cellular profile (30).

**Figure 2:**
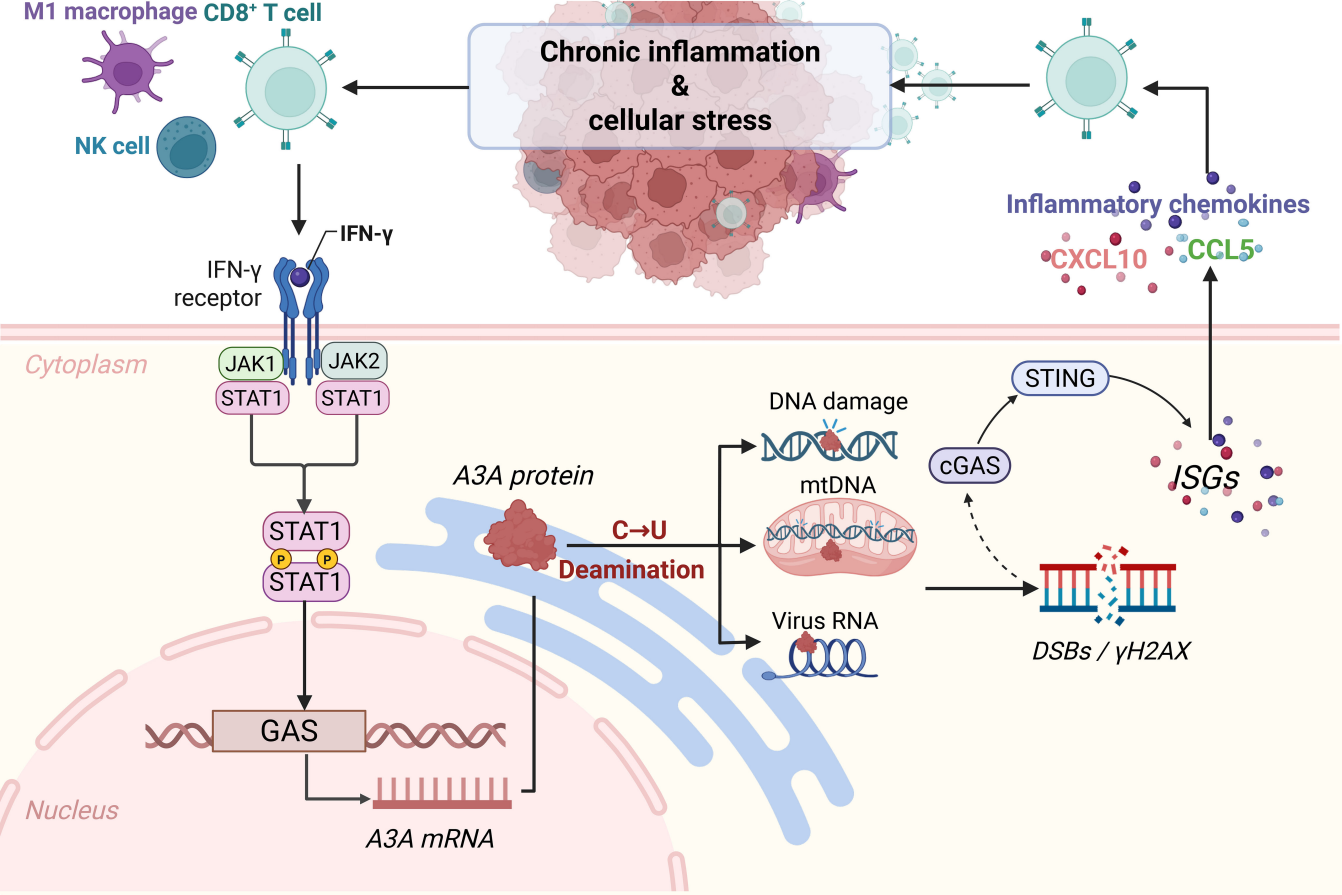
Positive feedback loop between inflammatory signaling, A3A activity, and cGAS–STING-driven immune activation. Chronic inflammation activates NK cells, CD8^+^ T cells, and M1-polarized macrophages to secrete IFN-γ, which signals through IFN-γR–JAK1/JAK2 to activate STAT1 and induce A3A transcription via GAS elements. Elevated A3A drives C→U deamination on exposed single-stranded nucleic acids (primarily ssDNA), promoting nuclear/mitochondrial DNA damage (e.g., DSBs and γH2AX) and cytosolic DNA accumulation that activates cGAS–STING, ISG programs, and chemokines (e.g., CXCL10 and CCL5). These signals recruit and activate additional effector lymphocytes, reinforcing IFN-γ production and sustaining the IFN-γ–STAT1–A3A axis. The figure was created with BioRender. DSBs, double-strand breaks.

At the regulatory level, chronic inflammatory signals are major upstream drivers of APOBEC3s expression. Cytokines such as IFN-γ, IL-6, and TNF-α released by infiltrating immune cells can induce A3A and A3B through interferon–JAK–STAT and NF-κB-dependent pathways, and APOBEC3A-mediated C→U RNA editing in myeloid cells in turn amplifies pro-inflammatory gene programs, thereby establishing a positive feedback loop between inflammatory signaling and APOBEC3s activity (36, 63). The integrated model of this interferon–A3A–cGAS–STING feedback loop is shown in **Figure 2**. In addition, hypoxia can increase genomic instability and alter patterns of tumor immune infiltration by upregulating A3B while simultaneously promoting A3A- and A3G-mediated RNA editing, which may help establish stress-adaptive programs in NK cells and CD8^+^ T cells (74, 80, 96).

Beyond tumor cells, immune cell-intrinsic APOBEC3s programs may shape differentiation and effector functions in a subset-selective manner (16, 45, 77). Among these, A3A provides a representative myeloid-centered example. In the myeloid compartment, A3A-mediated C-to-U RNA editing is broadly induced during M1 polarization and in monocytes/macrophages responding to hypoxia and interferon cues, where it promotes pro-inflammatory, M1-like programs; conversely, A3A loss dampens inflammation-driven M1 polarization and can shift cells toward M2-like features (81, 97). Consistent with this pro-inflammatory bias, A3A is enriched in monocytes and may also influence the differentiation of classical monocytes toward FCGR3A^+^ subsets (98).

Extending this concept to antigen-presenting lineages, inflammatory cytokines and interferons (e.g., IFN-α/TNF-α) can upregulate multiple members of the APOBEC3s in monocyte-derived dendritic cells, consistent with an innate “restriction/stress” module that could plausibly intersect with dendritic cell (DC) activation and T-cell priming in inflamed TIMEs (20, 22, 78). In NK cells, A3G has been linked to the modulation of NK responses, and under hypoxia or mitochondrial respiratory inhibition, NK cells exhibit A3G-mediated RNA editing, suggesting a role in stress-adaptive programs (80, 99). Downstream in T-cell differentiation, infectious disease studies have shown subset-biased A3G expression in CD4^+^ T cells (higher in Th1 than Th2) and implicate A3G in restricting HIV infection (100).

Notably, APOBEC3s activity can also engage stress–immune signaling beyond immune lineages. In human gastric epithelial organoid models, A3A activation can directly trigger intracellular stress and immune signaling and upregulate inflammation- and immunity-related genes such as *CXCL8*, *CCL20*, and *CDKN1A*, while the resulting DNA damage may be constrained by low-fidelity repair pathways, which together produce a persistent, immunostimulatory mutational burden (82). Neoepitopes generated by APOBEC3s-induced mutations can bind diverse MHC molecules, broaden the spectrum of T-cell recognition, and reduce opportunities for immune escape (101). In addition, A3G participates in DNA damage repair and supports cell survival, and the inhibition of its activity may increase sensitivity to radiotherapy and chemotherapy (38, 40).

Taken together, these observations indicate that APOBEC3 enzymes contribute to tumor biology beyond mutational accumulation within cancer cells. APOBEC3s activity can remodel the TIME by influencing immune cell differentiation, antigen presentation, and effector function. Large-scale clinical genomics studies have supported this view: in whole-genome data from 2,445 breast cancers, APOBEC-associated mutational signatures (SBS2 and SBS13) mark tumors with higher mutational burden and greater immune plasticity but also independently predict poor survival (102). Along with structural variant load and TP53-driven events, SBS2/13 defined a high-risk genomic subtype.

Thus, APOBEC3s activity acts as a double-edged sword between immune pressure and tumor evolution. In the short term, APOBEC3s may increase tumor immunogenicity and promote T cell-mediated control. Over longer time scales, the same activity can fuel genomic instability, clonal diversification, and eventual immune escape. Clinical data from multicenter NSCLC cohorts highlight this balance. In patients treated with immune checkpoint inhibitors (ICIs), sustained immune activation, reflected by a low neutrophil-to-lymphocyte ratio, early immune-related adverse events, and prolonged ICI exposure, is associated with durable T-cell responses and improved outcomes (83), illustrating how enhanced immunogenicity can coexist with adaptive resistance. This clinical pattern parallels the context-dependent roles of APOBEC3s in simultaneously promoting immunogenicity and enabling tumor adaptation.

To date, there is little evidence that APOBEC3 enzymes preferentially introduce mutations in canonical T-cell exhaustion genes (such as PDCD1, CTLA4, HAVCR2, or LAG3) or that they directly reprogram their transcription. Instead, most links between APOBEC3 activity, T-cell exhaustion, and immune escape are indirect: APOBEC3-high tumors tend to exhibit increased tumor mutational burden, chronic type I interferon and cGAS–STING signaling, and upregulation of PD-L1/PD-1 axes, features consistent with late-stage immune editing and dysfunctional T-cell states. Thus, whether APOBEC3s can directly rewire exhaustion programs remains an important open question.

Given that immunotherapy efficacy arises from the interplay between tumor genomics and the immune ecosystem, the systematic dissection of APOBEC3 regulatory networks and immune effects will be critical for refining our understanding of response and resistance and for guiding future combinatorial strategies.

## 5 APOBEC3s in immunotherapy response and resistance

ICIs disrupt the interaction between PD-L1 on tumor cells and PD-1 on T cells, thereby relieving inhibitory signaling and reinvigorating antitumor T-cell function. Clinical benefit from ICIs is strongly influenced by the efficiency of antigen presentation, the extent of immune activation, and the persistence of effector T-cell responses within the tumor immune ecosystem (103). APOBEC3s can increase tumor immunogenicity by inducing missense mutations and promoting neoantigen formation and thus may shape clinical responses to immune checkpoint blockade (ICB) (101).

Tumor mutational burden is an important biomarker for predicting ICI efficacy. APOBEC-associated mutations represent one of the major sources of tumor mutational burden in pan-cancer (84, 93, 94). Tumor neoantigens or neoepitopes arise from somatic missense mutations in cancer cells. These mutated peptides are presented on the cell surface by major histocompatibility complex molecules and are recognized by T cells as foreign antigens, which triggers immune activation. Pan-cancer analyses show that tumors with high APOBEC activity often display CD8^+^ T-cell enrichment, upregulation of CXCL9 and CXCL10, and increased PD-L1 expression, features that are typical of an inflamed and immune-active tumor microenvironment (48, 58, 93).

APOBEC-driven high mutational burden is closely linked to IFN-γ-driven immune activation. IFN-γ released by tumor-infiltrating T cells and NK cells not only enhances CXCL9 and CXCL10 expression and recruits additional effector lymphocytes (58, 98, 104) but can also drive adaptive immune resistance by upregulating PD-L1 on both tumor and non-malignant TIME cells (58, 105, 106). In parallel, this IFN-γ-driven, sustained antigenic and inflammatory pressure is accompanied by increased expression of inhibitory receptors on immune effectors (e.g., CTLA-4 and TIGIT on activated/exhausted T and NK cells), together with upregulation and engagement of their ligands (CD80/CD86 predominantly on antigen-presenting cells (APCs); CD155/CD112 on tumor cells and/or APCs) (58, 105, 106). Thus, APOBEC-associated neoantigens can enhance CD8^+^ infiltration and IFN-γ signaling, yet the same milieu amplifies checkpoint circuits that promote dysfunction and immune escape, supporting tumor persistence under immune pressure (58, 106–108).

Notably, T-cell exhaustion may create feedback to amplify APOBEC programs in tumor cells. Exhausted T cells lose cytotoxic capacity under chronic antigen exposure, yet can still sustain TNFα and IFN-γ signaling within the TIME. In an experimental setting, suboptimal immune pressure induced an APOBEC-associated mutator phenotype in tumor cells through a TNFα–PKC-dependent pathway, facilitating immune escape (109). Moreover, IFN-γ can upregulate A3B/A3G expression, providing an additional route to enhance APOBEC activity (79). Clinically, APOBEC3s signatures often correlate with PD-L1 expression, CD8^+^ T-cell infiltration, and responses to PD-(L)1 blockade across multiple tumor types (43, 61, 69, 76), suggesting a potential feedback loop coupling immune dysfunction to continued APOBEC3s activity.

Preclinical models further illustrate this dual role in treatment response and resistance. APOBEC3s-driven kataegis is tightly associated with mutations in key genes such as *ESR1*, *PIK3CA*, and *RB1* in breast cancer, and these alterations promote the emergence of resistant clones under immune or drug pressure (85). In mouse melanoma models, A3B overexpression increases sensitivity to ICIs but at the same time enhances resistance to chemotherapy (101). In HNSCC and other epithelial tumors, APOBEC-associated mutational signatures are closely linked to IFN-γ-driven inflammatory gene programs, PD-L1 upregulation, and increased neoantigen load, features that define immune-inflamed phenotypes and are associated with improved responses to PD-1/PD-L1 blockade, suggesting that APOBEC3 activity may help identify immunotherapy-sensitive subsets (76, 93, 94). Conversely, in NSCLC, APOBEC3s acts as a major driver of resistance to epidermal growth factor receptor tyrosine kinase inhibitor (EGFR-TKI) therapy (110). These observations highlight the evolutionary adaptation of APOBEC3s activity under therapeutic and immune pressure, with short-term gains in immunogenicity but long-term promotion of therapeutic resistance.

APOBEC3s activity also connects to immune phenotypes across cancer types. In HNSCC, both HPV-positive and HPV-negative subtypes show increased APOBEC3 gene expression and an immunoinflammatory phenotype. This pattern suggests that APOBEC3s activity may be an intrinsic driver of the immune-inflamed phenotype in HNSCC, independent of viral infection (111, 112). Emerging single-cell and spatial transcriptomic studies have suggested that, in some tumor types, APOBEC3-high regions may coincide with immune-active or inflamed niches, although this association still requires systematic validation (48). Clinically, breast tumors with a high APOBEC mutational burden can show better ICI response rates even when PD-L1 expression is low or tumor-infiltrating lymphocytes are scarce, suggesting that APOBEC-based metrics may complement traditional PD-L1 and tumor-infiltrating lymphocytes (TIL) markers (113). In a large multi-cohort analysis of 3,969 breast tumors, approximately 5% were hypermutated—enriched in metastatic disease—and approximately 60% of these hypermutated cases were dominated by APOBEC-associated signatures, which were linked to higher predicted neoantigen load and cytolytic activity. Notably, three patients with hypermutated breast cancer, including two with dominant APOBEC signatures, derived durable clinical benefit from pembrolizumab-based regimens (94). By contrast, in advanced urothelial carcinoma, high tumor mutational burden and APOBEC-associated mutational signatures are associated with a more favorable overall prognosis yet function as negative predictive markers for response to platinum-based chemotherapy, and their relationship with immune checkpoint inhibitor efficacy was not statistically significant in a small cohort, underscoring the need for validation in larger clinical studies (69).

Cross-cancer analyses identify immune-activated subtypes enriched for APOBEC3-associated mutational signatures in HNSCC, advanced urothelial carcinoma, NSCLC, pulmonary neuroendocrine tumors, ovarian clear cell carcinoma, esophageal adenocarcinoma, and breast cancer, typically showing increased immune cell infiltration and better responses to immunotherapy (61, 69, 73, 86, 87, 113–115). Nevertheless, the immunologic impact of APOBEC3 activity is context-dependent: in lung adenocarcinoma, it correlates with reduced immune infiltration, whereas in HNSCC and bladder cancer, it associates with immune activation (116).

Finally, APOBEC3s-induced mutational spectra hold promise as tools for immunotherapy stratification and combination therapy design (88, 93, 117). The synthetic lethality potential between APOBEC3s activity and DNA repair defects, such as HR deficiency (HRd) or NHEJ deficiency (NHEJd), provides a conceptual basis for regimens that combine poly (ADP-ribose) polymerase (PARP) inhibition with PD-1 blockade (73).

However, the mechanistic links between APOBEC3 activity, the repair or misrepair of deamination lesions by specific DNA repair pathways, and downstream cGAS–STING–interferon signaling remain incompletely defined; these context-dependent circuits likely determine the balance between neoantigen formation, checkpoint engagement, and T-cell function and should guide future strategies that integrate genomic targeting with immunomodulation.

## 6 Current therapeutic strategies and emerging opportunities involving APOBEC3s

APOBEC3s is now recognized as a central regulator that links genome dynamics to immune surveillance (118–120). Its activity is episodic, context-dependent, and shaped by inflammatory cues, DNA repair capacity, and microenvironmental signals. In future work, the focus should shift from a simple “on–off” view to spatiotemporal control of APOBEC3s, with the aim of exploiting their immunogenic potential while limiting long-term genomic damage.

At the mutational level, APOBEC3s generates highly stereotyped signatures. Neural embedding models such as mutational embeddings for signatures identification in clinical assays (MESiCA) can detect these signatures with high accuracy, even when only one to 15 single-nucleotide variants (SNVs) are present (110). This stability provides a quantitative readout for monitoring tumor evolution and remodeling of the immune microenvironment (86, 110, 119, 120). Pharmacological and genetic strategies are being developed to modulate APOBEC3 activity (121).

Small-molecule inhibitors, RNA interference, and gene-editing tools have been tested in preclinical models (45, 121). Among direct approaches, the clearest progress is for A3A, where structure-guided hairpin ssDNA inhibitors bearing 2′-deoxy-5-fluorozebularine competitively trap the catalytic center and are supported by high-resolution structural evidence (31, 45, 121). Because delivery and nuclease susceptibility are major liabilities for oligonucleotide inhibitors, next-generation designs have been optimized for potency and nuclease stability and extended to additional isozymes (including A3G), addressing a key translational bottleneck for this inhibitor class (122). Early small-molecule efforts aimed at APOBEC family catalytic pockets have also been reported, but achieving drug-like potency and isozyme selectivity remains difficult given the conserved zinc-dependent deaminase fold and shared substrate-recognition principles across family members (31).

Accordingly, indirect pharmacologic downregulation may be the most immediately tractable route in some settings, exemplified by sulforaphane suppressing the lncRNA H19/A3G axis and inhibiting pancreatic ductal adenocarcinoma growth *in vivo* (123). Targeted microRNA degradation represents a complementary approach. RNA-based triggers can selectively eliminate oncogenic miRNAs, indirectly reshaping gene networks connected to APOBEC pathways (124, 125).

Across these modalities, the key practical question is not only which APOBEC3 member to modulate but also when, so integrating APOBEC-signature dynamics, as pharmacodynamic readouts and aligning intervention with periods of therapy-/inflammation-linked mutational bursts should be prioritized for clinical translation.

Molecular engineering provides an additional level of control. Artificial intelligence-guided redesign of A3A in the Professional APOBECs platform allows precise RNA C→U editing with low off-target activity (126). *In vivo*, this system can modulate PCSK9 and Mef2c transcript levels and improve metabolic and neurological phenotypes (126). Recent work further suggests that A3C may function as a broadly expressed, relatively “mild” regulator of baseline genomic stability. A3C is enriched in highly transcribed regions, appears to depend on transcription-associated ssDNA exposure, and is rarely hyperactivated in tumors (39). These features make A3C an attractive scaffold for low off-target RNA editing and for protective roles in steady-state mutational control and antiviral defense (39, 126). Together, these studies have indicated that APOBEC3s can be repurposed from endogenous mutators into programmable effectors at both the DNA and RNA levels.

Beyond direct inhibition, APOBEC-driven mutagenesis can be leveraged therapeutically via two complementary axes: immunogenic neoantigen generation and heightened dependence on DNA repair and replication stress pathways. APOBEC-induced mutations generate heteroclitic neoepitopes that broaden T-cell recognition and can support the design of next-generation cancer vaccines and adoptive T-cell strategies (86, 101, 119). Clinical sequencing in NSCLC has shown that many acquired MET kinase-domain mutations and resistance lesions to EGFR or ALK inhibitors carry SBS2/SBS13 signatures (127, 128). Modeling studies have predicted that complete inhibition of A3A and A3B could delay resistance by 0%–1,290% and that the contribution of APOBEC signatures correlates linearly with the delay in resistance (127, 128). These data support a strategy of using APOBEC inhibition to postpone resistance while maintaining antitumor immunity.

Oncolytic virotherapy highlights the plasticity of APOBEC-mediated immune modulation. Overexpression of A3B can remodel the tumor immunopeptidome and induce mutant CSDE1 peptides that act as heteroclitic neoepitopes (101, 119). These epitopes elicit CD8^+^ T-cell responses and allow cross-recognition of mutant and wild-type peptides, enabling bystander clearance of non-mutant tumor cells. Adoptive transfer of T cells specific for A3B-induced epitopes can control tumor growth and generate durable memory, leading to the concept of heteroclitic epitope-activated therapy (86, 101, 119). Oncolytic viruses can be integrated directly into this framework. VSV-IFNβ treatment activates A3B, induces characteristic C→T mutations, and produces recurrent CSDE1 alterations such as CSDE1^P5S^ (129). These changes confer resistance and restrict viral replication but also create escape-associated tumor antigens that remain immunogenic (129). Based on these findings, a “trap-and-ambush” model has been proposed. In the trap phase, oncolytic virus therapy drives A3B-dependent mutagenesis. In the ambush phase, vaccines or epitope-specific T cells target the newly formed antigens and eradicate escape subclones. A sequential dual-virus regimen (VSV-IFNβ followed by VSV-IFNβ-P/M), combined with CD200 checkpoint blockade, enhances reinfection of mutant subclones, boosts antitumor T-cell responses, and improves long-term cure rates (129). These results suggest that APOBEC3-induced mutations can serve as predictable, “designed” targets for oncolytic virus-based immunotherapy and for rational combination with immune checkpoint inhibitors.

APOBEC-induced cytidine deamination provides a synthetic lethality lever. APOBEC3 mutagenesis often occurs in bursts under chemotherapy, radiotherapy, or immune checkpoint blockade and is tightly coupled to replication stress (34, 66, 86). In tumors with HRd or NHEJd, APOBEC-induced lesions accumulate as persistent damage (38, 40, 66, 130). PARP inhibitors block single-strand break repair in HRd cells, while ATR inhibitors blunt the replication stress response, allowing APOBEC-mediated damage to persist (66). Pan-cancer analyses show frequent co-occurrence of APOBEC signatures with HRd or NHEJd and indicate that A3G can facilitate double-strand break repair (38, 40, 130). Inhibiting A3G increases sensitivity to radiotherapy and PARP inhibition, revealing exploitable repair dependencies. Treatment pressure can further induce A3B, accelerating subclonal evolution and resistance; targeted A3B inhibition, alone or in combination with NF-κB inhibition or checkpoint blockade, shows synergistic effects in preclinical models (41, 86, 119). In multiple myeloma, a high SBS2/SBS13 burden associates with increased progression and relapse, and APOBEC3 activation correlates with immune remodeling, particularly NK-cell responses, in patients treated with CD38 monoclonal antibody combinations (119). These observations support incorporating APOBEC-targeted agents into synthetic-lethal and immunomodulatory strategies that combine DNA-damaging therapies with PARP/ATR inhibition and immunotherapy.

Finally, systems and spatial immunology approaches are essential to place APOBEC3 biology in tissue context. Single-cell and spatial multi-omics can map APOBEC3 expression trajectories across tumor cells, myeloid, and lymphoid compartments and relate it to DNA damage markers (γH2AX and pRPA) and local immune architecture; combined with whole-genome sequencing, these data directly link APOBEC mutational signatures to cellular states, replication timing, and microenvironmental cues (93, 112, 131–133).

Beyond the well-characterized A3A/A3B axis, recent breast cancer work implicates A3C as a regulator of precancerous micromutation patterns, with a distinct spectrum enriched in highly transcribed regions and dependent on transcription-associated ssDNA exposure (116). A3C is broadly expressed in normal tissues and rarely hyperactivated in tumors, suggesting protective roles in basal mutational control, RNA editing, and antiviral defense (131). These features make A3C an attractive scaffold for low off-target RNA-editing tools and raise the possibility that “protective” APOBEC3 members could be harnessed therapeutically, rather than merely suppressed. Accordingly, APOBEC-enriched tumors may be rational candidates for integrated regimens that pair immune activation with DDR-targeting agents, guided by biomarker frameworks incorporating APOBEC-signature burden, APOBEC3 isozyme expression, DNA-repair deficiency, replication stress/DNA damage readouts, and immune-inflamed features. Future work should focus on spatiotemporal tuning of APOBEC3 activity to capture short-term gains in immunogenicity while limiting long-term genomic instability and therapeutic resistance.

## 7 Conclusions

APOBEC3 enzymes sit at a critical intersection between genomic instability and tumor–immune interactions. By inducing context-specific cytosine deamination, they drive somatic mutagenesis and clonal evolution while simultaneously expanding the neoantigen repertoire and activating innate immune sensing pathways. This dual role means that APOBEC3 activity can both facilitate immune escape and, under certain conditions, enhance antitumor immunity and responsiveness to checkpoint blockade.

Moving forward, incorporating APOBEC3 expression levels and mutational signatures into molecular profiling could clarify when these enzymes act mainly as tumor-driving mutators and when they instead generate exploitable immunogenic stress. Rational strategies that either restrain excessive APOBEC3-driven instability or exploit APOBEC-derived neoantigens in combination with targeted and immune-based therapies have the potential to deliberately reshape tumor–immune equilibrium and improve precision immuno-oncology.

## Glossary

AID: activation-induced deaminase
APOBEC: The apolipoprotein B mRNA editing catalytic polypeptide-like
APOBEC3A: A3A
APOBEC3B: A3B
APOBEC3C: A3C
APOBEC3F: A3F
APOBEC3G: A3G
APOBEC3H: A3H
APOBEC3s: APOBEC3 subfamily
HNSCC: head and neck squamous cell carcinoma
HR: homologous recombination
HRd: homologous recombination deficiency
ICB: immune checkpoint blockade
ICI: immune checkpoint inhibitor
NHEJ: non-homologous end joining
NHEJd: non-homologous end joining deficiency
NSCLC: non-small cell lung cancer
TIME: tumor immune microenvironment
